# Broadly neutralizing antibody responses in a Chinese acute HIV-1 infection cohort of men who have sex with men

**DOI:** 10.1371/journal.ppat.1013822

**Published:** 2026-01-02

**Authors:** Linhong Yao, Yang Gao, Wen Tian, Haibo Ding, Hong Shang, Xiaoxu Han

**Affiliations:** 1 State Key Laboratory for Diagnosis and Treatment of Infectious Diseases, NHC Key Laboratory of AIDS Prevention and Treatment, National Clinical Research Center for Laboratory Medicine, The First Hospital of China Medical University, China Medical University, Shenyang, China; 2 Key Laboratory of AIDS Immunology, Chinese Academy of Medical Sciences, Shenyang, China; 3 Key Laboratory of AIDS Immunology of Liaoning Province, Shenyang, China; 4 Collaborative Innovation Center for Diagnosis and Treatment of Infectious Diseases, Hangzhou, China; University of Illinois at Chicago College of Medicine, UNITED STATES OF AMERICA

## Abstract

Numerous broadly neutralizing antibodies (bnAbs) have been isolated from individuals with chronic HIV-1 infection, yet eliciting bnAbs through active immunization remains challenging. Investigating naturally infected patients whose plasma exhibits broadly neutralizing activity may reveal the factors driving bnAb development and inform vaccine design. We analyzed the clinical, immunological, and virological correlates of bnAb responses in a longitudinally followed, antiretroviral therapy (ART)-naïve, acute HIV-1 infection (AHI) cohort (n = 52) of men who have sex with men (MSM) in Shenyang, China. Neutralizing activity was assessed in participant plasma samples collected at the last available time point (LTP), prior to ART initiation, loss to follow-up, or the study data cutoff (median: 3.82 years, range: 3.14–4.82 years). To determine the occurrence and timing of HIV-1 multiple infection, we amplified and deep-sequenced the *env* C2–V4 and *pol*-RT regions (~450 bp) from plasma collected at baseline, one year, two years, and LTP. Individuals with multiple infection developed significantly stronger bnAb responses at LTP than those with monoinfection. Notably, acquisition of a second HIV-1 strain within or beyond one year after primary infection was associated with enhanced bnAb responses, with a higher odds ratio (OR) observed for superinfection occurring beyond one year. These findings indicate the potential role of immunogen diversity and immunization timing in bnAb induction, supporting vaccine strategies that mimic delayed sequential antigen delivery.

## Author summary

Understanding the processes that drive the development of broadly neutralizing antibodies (bnAbs) during natural HIV-1 infection is crucial for effective vaccine design. In this study, we investigated clinical, immunological, and virological factors associated with bnAb responses in a cohort of men who have sex with men (MSM) with acute HIV-1 infection (AHI) in Shenyang, China. We found that individuals with multiple infection exhibited stronger bnAb responses, regardless of whether the second strain was acquired within or beyond one year after primary infection, with a greater effect in the latter group. These findings suggest that delayed sequential exposure to diverse HIV-1 strains may promote bnAb development and inform future vaccine strategies.

## Introduction

Human immunodeficiency virus (HIV) exhibits an extraordinary replication capacity and mutation rate [1], posing significant challenges to vaccine development. Broadly neutralizing antibodies (bnAbs) neutralize diverse HIV-1 subtypes by targeting semi-conserved regions of the envelope glycoprotein: V1V2 apex, V3-glycan site, CD4 binding site (CD4bs), gp120-gp41 interface, gp120 silent face, gp41 fusion peptide, and the membrane-proximal external region (MPER) [2]. Consequently, eliciting bnAbs has become a major goal in HIV vaccine design. Recent animal studies and clinical trials have demonstrated the successful induction of rare bnAb precursors following vaccination, representing a critical advancement in the field [3,4]. However, the maturation of these precursors into potent bnAbs remains poorly understood. This can largely be attributed to the complex regulation of bnAb development by host factors, immune environment, and viral characteristics [5].

Broadly nAb responses usually develop 2–4 years after HIV-1 infection and are detectable in approximately 10–30% of infected individuals. Several factors have been associated with their development, including low CD4^+^ T-cell counts, specific HLA genotype, prolonged infection duration, viral subtype, high viral load, and exposure to HIV-1 multiple infection [6–12]. Among these, multiple infection has gained increasing attention for its potential role in promoting cross-reactive antibody responses. Multiple infection refers to the acquisition of genetically distinct HIV-1 strains, either simultaneously (coinfection) or sequentially (superinfection). Although most cases involve two strains (dual infection), rare instances of superinfection with three strains have been reported [13–17]. These exposures may functionally mimic multivalent or prime-boost immunization strategies and provide important insights for bnAb-based vaccine design.

To better understand the determinants of bnAb responses, we analyzed clinical, immunological, and virological factors associated with bnAb development in a cohort of men who have sex with men (MSM) with acute HIV-1 infection (AHI) from Shenyang, China, aiming to inform immunogen design and optimize vaccination regimens.

## Results

### Broadly neutralizing antibody responses in the AHI cohort

Fifty-two participants were enrolled from an AHI prospective cohort in Shenyang, China [17,18]. All participants were followed for over two years and characterized by demographics, HIV-1 subtype, CD4⁺ and CD8 ⁺ T-cell counts, viral load, and disease progression (Table 1). To evaluate humoral immune responses, we assessed the plasma neutralizing activity at each participant’s last available time point (LTP) prior to antiretroviral therapy (ART) initiation, loss to follow-up, or the study data cutoff (median duration of infection: 3.82 years; range: 3.14–4.82 years). Neutralization assays were performed using a standardized global panel of 12 Env-pseudotyped tier 2 viruses representing major HIV-1 clades (A, B, C, G) and circulating recombinant forms (CRF01_AE, CRF07_BC, AC) (S1A Fig in S1 Text) [19–23].

**Table 1:** Characteristics of AHI cohort participants by neutralizing antibody response.

Characteristics	Broad neutralizers	Non-broad neutralizers	*P* value^a^
	n = 20	n = 32	
**Age-EDI, years**			0.631
Median (IQR)	32.50 (27.75, 39.00)	30.50 (25.00, 38.25)	
**Duration of infection, years**			0.003
Median (IQR)	4.72 (3.33, 6.22)	3.45 (3.13, 4.36)	
**CD4** ^** +**^ ** T-cell count, cells/μl**			0.617
Mean (SD)	336.94 (174.14)	312.61 (161.40)	
**CD4** ^** + **^ **/CD8** ^** +**^ ** T-cell ratio**			0.598
Mean (SD)	0.40 (0.22)	0.36 (0.19)	
**Viral load, log10 copies/ml**			0.901
Mean (SD)	4.54 (0.56)	4.52 (0.75)	
Missing, %	0 (0%)	1 (3.13%)	
**Disease progression, %**			0.747
RP	4 (20.00%)	8 (25.00%)	
TP	16 (80.00%)	24 (75.00%)	
**Primary infection subtype, %**			0.378
CRF01_AE	13 (65.00%)	25 (78.13%)	
CRF07_BC	4 (20.00%)	2 (6.25%)	
others	3 (15.00%)	5 (15.63%)	
**Infection type, %**			0.003
monoinfection	10 (50.00%)	29 (90.63%)	
multiple infection	10 (50.00%)	3 (9.38%)	
**Time of second HIV-1 strain acquisition, %**			0.002
monoinfection	10 (50.00%)	29 (90.63%)	
0–1 year	4 (20.00%)	2 (6.25%)	
>1 year	6 (30.00%)	1 (3.13%)	

^a^*P* values were calculated using the Mann–Whitney U test for continuous variables (median [IQR]), the t-test for normally distributed variables (mean [SD]), and Fisher’s exact test for categorical variables due to small expected frequencies.

Broad neutralizers: neutralization score ≥ 0.5; non-broad neutralizers: neutralization score < 0.5.

EDI: estimated date of infection; RP: rapid progression; TP: typical progression; IQR: interquartile range; SD: standard deviation.

**Fig 1:**
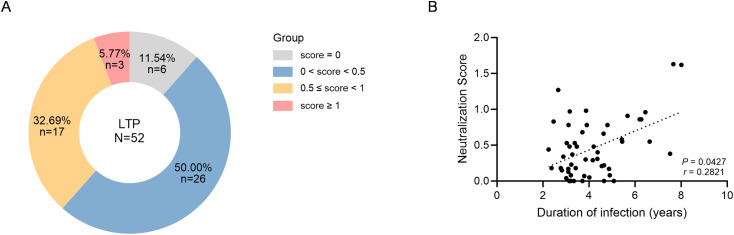
Neutralizing antibody responses in the AHI cohort. **(A)** Distribution of participants across neutralization score categories at last available time point (LTP) prior to antiretroviral therapy (ART) initiation, loss to follow-up, or the study data cutoff, shown as a hollow donut chart. **(B)** Association between duration of infection and neutralization scores. Dashed black line indicates linear regression fit.

A neutralization score was calculated for each sample to reflect both breadth and potency as previously described (S1B Fig in S1 Text) [7,24,25]. 38.46% (20/52) of participants demonstrated broad neutralization (score ≥ 0.5), corresponding to a neutralization breadth ≥ 50% or a geometric mean titer (GMT) ≥ 60. Among these, 15.00% (3/20) achieved elite neutralization (score ≥ 1.0), corresponding to a neutralization breadth ≥ 67% and GMT ≥ 100 (Fig 1A, S1A Fig in S1 Text). Half of the participants maintained limited neutralization (0 < score < 0.5), whereas 11.54% (6/52) showed no detectable neutralizing activity (score = 0) (Fig 1A). Notably, neutralization scores were positively correlated with duration of infection (Spearman’s *P* = 0.0427, *r* = 0.2821; Fig 1B), consistent with the gradual development of bnAb responses over time.

### Multiple infection broadens and strengthens bnAb responses

**Fig 2:**
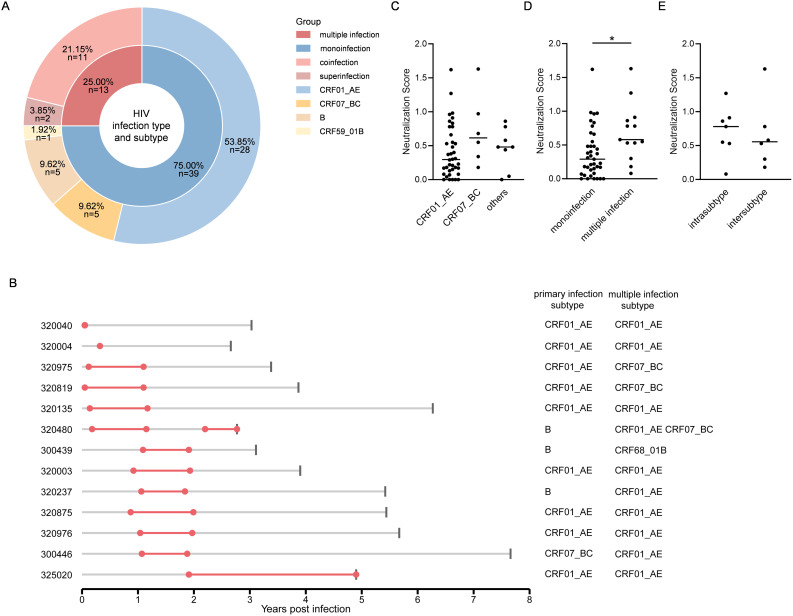
HIV-1 infection profiles and comparative analyses of bnAb responses across groups. **(A)** Nested donut chart showing the number and proportion of participants (n, %) by HIV-1 infection types and viral subtypes. **(B)** Timeline of viral infections in participants with multiple infection. Participant IDs are listed on the left. Gray horizontal lines indicate follow-up duration since primary infection, with gray vertical ticks marking LTP. Red line segments indicate the estimated multiple infection windows for the second and third strains. **(C-E)** Group comparisons of neutralization scores at LTP used the Kruskal–Wallis test (panel C, across primary infection subtypes) and Mann–Whitney U tests (panel D, between infection types; panel E, between multiple infection types). * *P* < 0.05.

To further explore whether viral exposure complexity influences bnAb development, we analyzed longitudinal HIV-1 sequences to track virus–antibody co-evolution. Building upon our previously established cohort framework with newly defined immunological endpoints [17], we amplified and deep-sequenced the *env* C2–V4 and *pol*-RT regions (~450 bp) from plasma collected at baseline, 1 year, 2 years, and LTP. Of the 52 participants, 39 were classified as monoinfected and 13 as multiply infected. CRF01_AE was the predominant subtype (40/52), followed by CRF07_BC. Participants with multiple infection included 2 coinfections and 11 superinfections, comprising 7 intrasubtype and 6 intersubtype events (Figs 2A–B, S2 Fig in S1 Text). Longitudinal sequence data enabled estimation of secondary strain acquisition timing: 5 cases occurred within 1 year, 6 between 1–2 years, and 1 beyond 2 years after primary infection; one individual (Participant ID: 320480) experienced triple infection, with superinfection events within 1 year and again beyond 2 years (Fig 2B).

We evaluated subtype specificity of the neutralization profiles. Neutralization scores did not differ by primary infection subtypes (Fig 2C), and stratification by subtypes detected during follow-up likewise showed no group-level advantage of subtype-matched versus unmatched panel viruses, although a small subset exhibited stronger matched responses (S3 Fig in S1 Text). Separately, bnAb responses developed in 76.92% (10/13) of participants with multiple infection, compared to 25.64% (10/39) of those with monoinfection. Neutralization scores were significantly higher in the multiple infection group (*P* < 0.05; Fig 2D), with no significant difference between intrasubtype and intersubtype cases (Fig 2E).

Overall, our findings suggest that individuals with multiple infection are more likely to develop broadly neutralizing activity.

### Timing of second HIV-1 strain acquisition correlates with bnAb responses

We next assessed whether the timing of second HIV-1 strain acquisition impacted bnAb development. Participants were stratified into broad or non-broad neutralizers based on a neutralization score threshold of 0.5. The distribution of second HIV-1 strain acquisition timing differed significantly between the two groups (*P* < 0.01, Table 1). In univariate analysis, acquisition of a second strain beyond one year after primary infection was significantly associated with enhanced bnAb responses (OR = 17.40, 95% CI: 1.75–172.62, *P* = 0.02; Table 2), while acquisition within one year showed a borderline association that did not reach statistical significance (OR = 5.80, 95% CI: 0.87–38.46, *P* = 0.07).

**Table 2:** Factors associated with bnAb responses, analyzed by logistic regression.

Characteristic	Univariable analysis		Multivariable analysis*	
	OR (95% CI)	*P* value	aOR (95% CI)	*P* value
**Age-EDI**	1.01 (0.95-1.07)	0.82	—	—
**Duration of infection**	1.81 (1.12-2.95)	0.02	1.84 (1.07-3.16)	0.03
**CD4 + T-cell count**	1.00 (1.00-1.00)	0.61	—	—
**CD4 + /CD8 + T-cell ratio**	2.21 (0.13-38.74)	0.58	—	—
**Viral load**	1.03 (0.44-2.44)	0.94	—	—
**Disease progression**				
TP	Ref		—	—
RP	0.75 (0.19-3.02)	0.68	—	—
**Primary infection subtype**				
CRF01_AE	Ref		—	—
CRF07_BC	3.85 (0.59-25.03)	0.15	—	—
others	1.15 (0.23-5.84)	0.86	—	—
**Time of second HIV-1 strain acquisition**				
monoinfection	Ref	—	Ref	—
0–1 year	5.80 (0.87-38.46)	0.07	9.37 (1.20-73.42)	0.03
>1 year	17.40 (1.75-172.62)	0.02	12.58 (1.16-136.86)	0.04

*In multivariable analysis, there was no significant interaction between duration of infection and time of second HIV-1 strain acquisition in predicting bnAb responses.

OR: odds ratio; CI: confidence interval; aOR: adjusted odds ratio

In a multivariable logistic regression model adjusting for duration of infection, acquisition of a second strain within one year became significantly associated with bnAb responses (adjusted OR [aOR] = 9.37, 95% CI: 1.20–73.42, *P* = 0.03), suggesting a potential confounding effect of infection duration. Acquisition beyond one year after primary infection remained statistically significant (aOR = 12.58, 95% CI: 1.16–136.86, *P* = 0.04). Collectively, these findings indicate that multiple infection enhances bnAb development, with a stronger effect observed when the second strain was acquired beyond one year after primary infection. In the sensitivity analysis, application of the Gaussian mixture model (GMM)-derived cutoff (0.48) resulted in no reclassification relative to the primary definition (S4 Fig in S1 Text), supporting the robustness of our findings.

## Discussion

We explored factors associated with bnAb responses during natural HIV-1 infection to provide empirical evidence for HIV immunization strategies. Our results highlight two key aspects: (i) multiple infection facilitates broadly neutralizing activity, supporting the design of multivalent antigen combinations; and (ii) second strain acquisition within or beyond 1 year after primary infection is associated with bnAb responses, with delayed acquisition beyond 1 year showing a stronger effect. These results may inform the immunization schedule by incorporating extended prime-boost intervals.

In our AHI cohort, 25% of participants experienced multiple infection. Regular sampling intervals and continuous viral sequence monitoring enabled us to precisely trace the timing of second strain acquisition and assess its relationship to bnAb development. Two mechanisms may underlie the observed association: (i) sustained stimulation of conserved epitopes shared across strains, promoting the maturation of existing bnAb lineages; and (ii) additive responses to strain-specific epitopes introduced by each infection, broadening the neutralization profiles [26].

Previous evidence linking multiple infection to bnAb responses has been inconsistent across cohorts, and the temporal dimension has not been systematically explored. In a Kenyan cohort of 12 superinfected women, most cases involved intra- or intersubtype superinfection with HIV-1 subtypes A, D, and C. These women developed stronger and broader bnAb responses, consistent with our findings. However, the wide range of superinfection timing (from < 2 months to 5 years after primary infection) restricted the ability to assess how timing influences bnAb development [10]. In a San Diego cohort of 10 intrasubtype B superinfection cases, most secondary infections occurred within 1 year, when humoral immunity is still maturing and vulnerability to superinfection may be increased. Antibody responses improved after superinfection, but bnAb development was rare; only one participant developed broad neutralization during follow-up, providing limited information on timing-related effects [27]. Our study confirmed the association between multiple infection and bnAb enhancement in a CRF-dominated epidemic setting, and extended previous work by revealing that both early (≤ 1 year) and delayed (> 1 year) secondary infection were linked to stronger bnAb responses, with delayed events showing greater effect estimates.

These findings underscore the importance of the timing of secondary antigen exposure in shaping bnAb development, offering insights relevant to the design of sequential immunization regimens. The subgroup with multiple infection was relatively small (n = 13), which limits statistical power and results in wide confidence intervals for timing comparison. Residual confounding by infection duration may still be present even after adjustment. Despite these limitations, this ART-naïve AHI cohort provides rare longitudinal data on natural bnAb evolution, which is becoming increasingly difficult to document under universal “treat all” ART guidelines. Furthermore, the identification of broad neutralizers from diverse infection types lays the foundation for future research on viral-antibody co-evolution and ultimately advances the rational engineering of next-generation HIV vaccine immunogens.

## Materials and methods

### Ethics statement

Ethical approval was obtained from the Ethics Committee of the First Affiliated Hospital of China Medical University (approval Nos. [2008]73, [2018]35, and [2023]605). All participants provided written informed consent before enrollment and sample collection. Clinical management, including ART initiation, followed national guidelines in effect at the time and was not altered for research purposes. After China adopted the “treat all” policy in 2016, ART was offered to all diagnosed individuals; only one participant voluntarily declined ART despite repeated counseling. All procedures complied with institutional and national ethical standards and with the principles of the Declaration of Helsinki and its later amendments.

### Study population

Fifty-two participants with AHI were enrolled between 2008 and 2014 through prospective follow-up of a high-risk MSM population at the First Affiliated Hospital of China Medical University [17,18], with follow-up continuing until April 2017. Inclusion criteria were: 1) the estimated time of HIV-1 infection was < 3 months; 2) a follow-up period > 2 years, during which longitudinal plasma samples, CD4 ⁺ T-cell counts, viral loads, and epidemiological data were collected; and 3) no initiation of ART during the study period. Infection dates were estimated by a standardized procedure [28,29]: participants were followed at 10-week serologic intervals to detect seroconversion; if a single unequivocal exposure was reported, that date was used and verified against the Fiebig stage at diagnosis; otherwise, the midpoint between the last seronegative and the first seropositive visit was used.

Additional details on the materials and methods, key reagents, and experimental procedures are provided in S2 Text.

## Supporting information

S1 Text Supplementary figures with corresponding legends. (DOCX)

S2 Text Supplementary experimental details. (DOCX)
