# Supplementary material for: Broadly neutralizing antibody responses in a Chinese acute HIV-1 infection cohort of men who have sex with men
Source: PLoS Pathog. 2026 Jan 2;22(1):e1013822. doi: 10.1371/journal.ppat.1013822 (PMC12758743; doi:10.1371/journal.ppat.1013822)
Supplement: S1 Text — (DOCX) [file ppat.1013822.s001.docx]

**
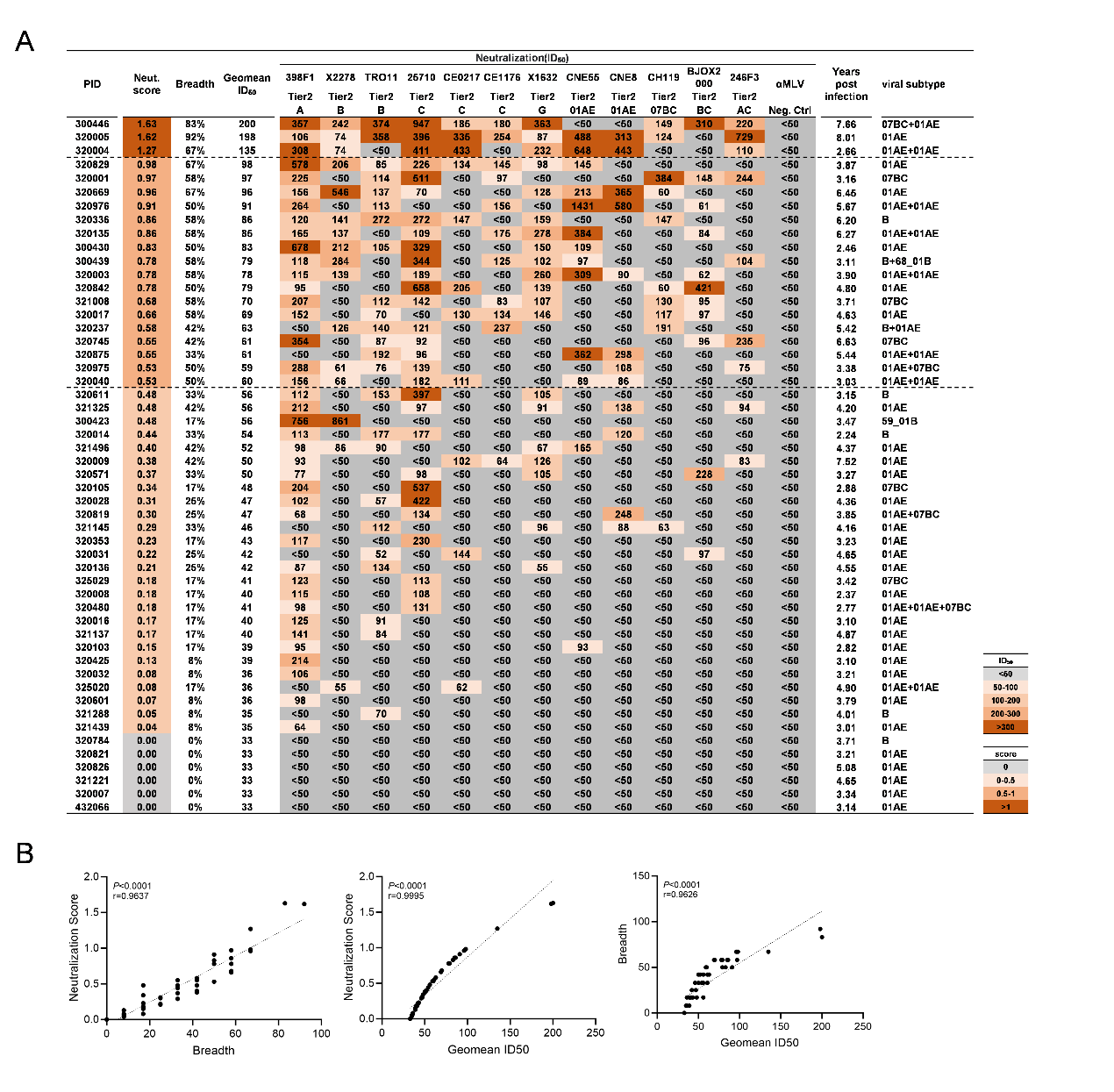
**

**S1 Fig. Neutralization profiles of plasma from 52 participants against a 12-virus global pseudovirus panel at LTP.** (A) Plasma samples collected at LTP from AHI participants were tested for neutralization against a standardized 12-virus global panel. The heatmap displays the raw neutralization ID₅₀ values. Neutralization score was calculated as the average of log-transformed ID₅₀ values [score = average (log3 (dilution/100) + 1)]. Neutralization breadth represents the percentage of viruses with ID₅₀ ≥ 50. Dashed lines distinguish broad neutralizers (score ≥ 0.5), elite neutralizers (score ≥ 1.0), and non-broad neutralizers (score < 0.5). (B) Pairwise Spearman’s rank correlations between neutralization score, breadth, and potency.

**
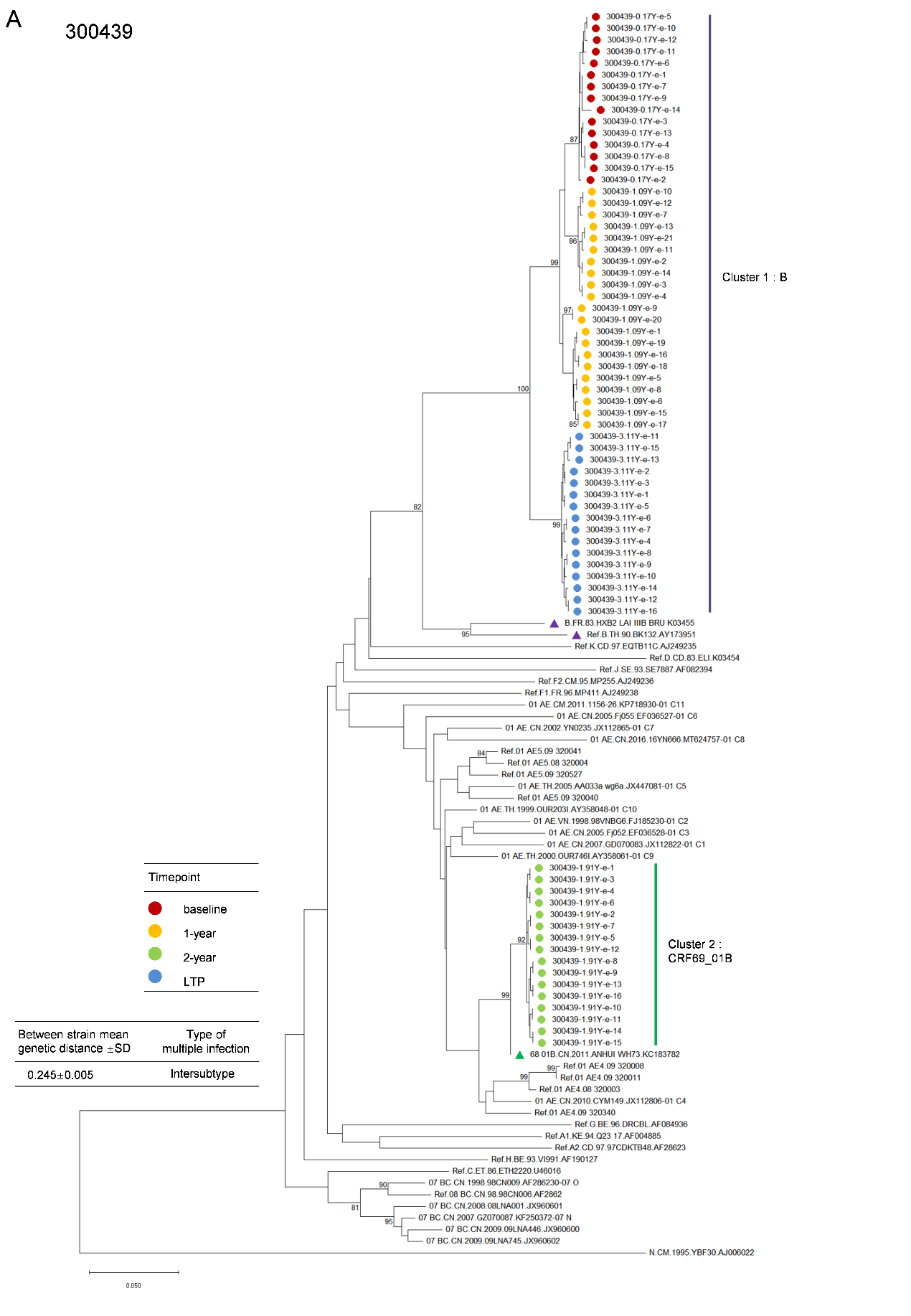

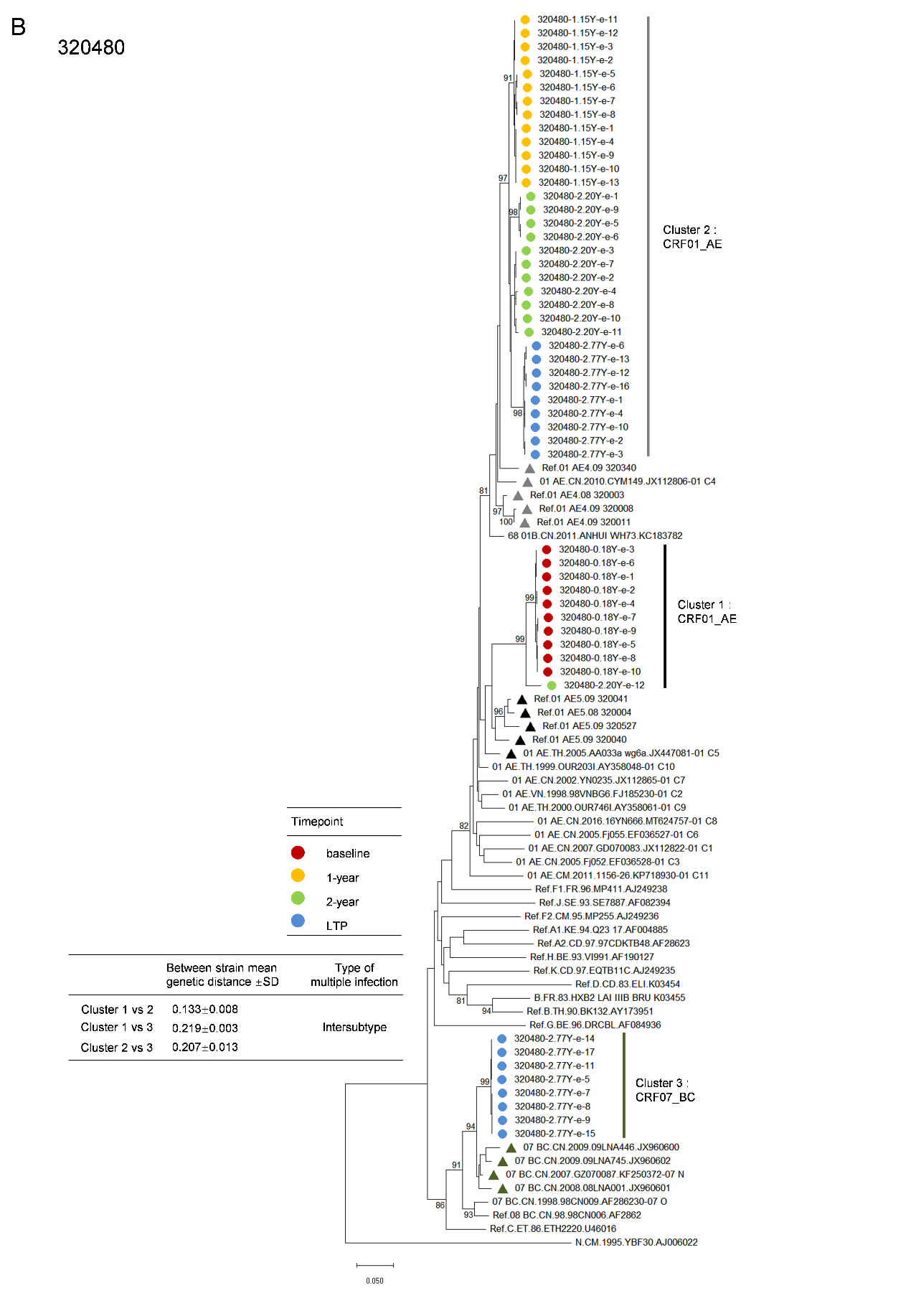

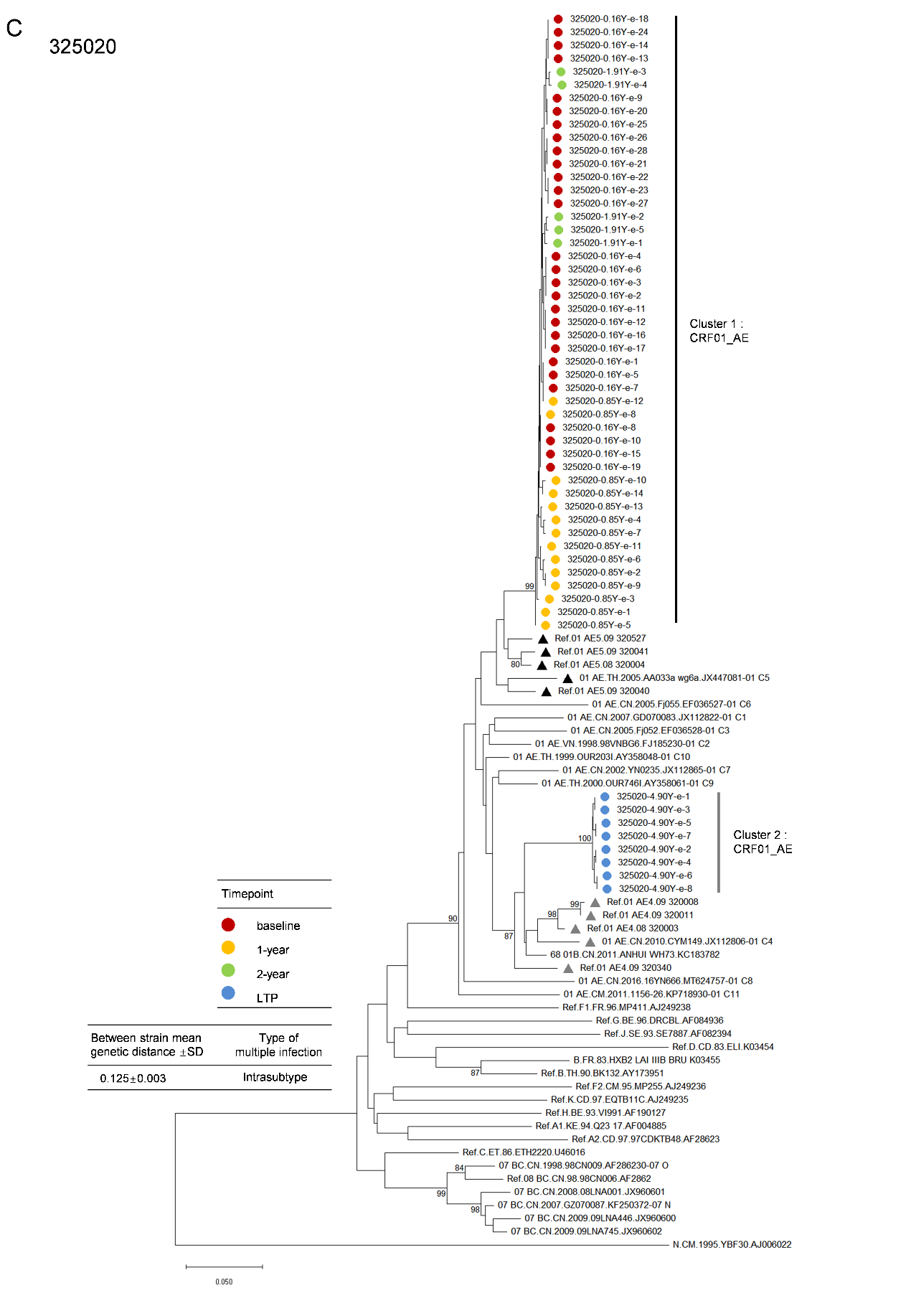

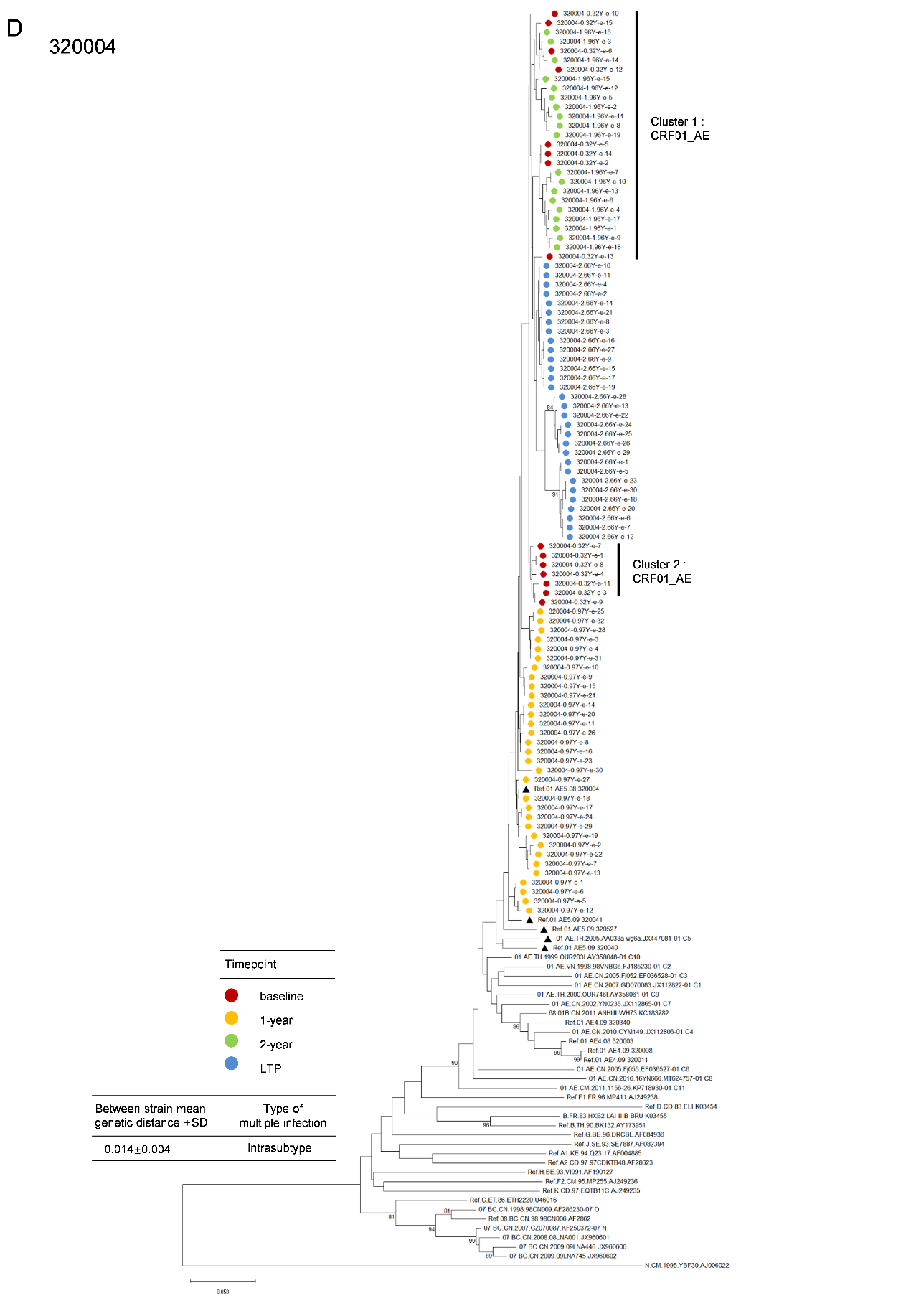
S2 Fig. Phylogenetic trees of longitudinal *env* sequences for participants with multiple infection.** Neighbor-joining trees were constructed using consensus env sequences from participants 300439 (A), 320480 (B), 325050 (C), and 320004 (D), along with reference sequences. Bootstrap support values > 80% (1,000 replicates) are shown at major nodes. Triangles indicate reference strains of the same subtype(s) as the identified infections, and solid circles denote participant-derived sequences sampled at different timepoints (color-coded as shown in the legend). Average pairwise genetic distances between viral clusters are summarized in the accompanying table. The remaining nine cases with multiple infection in this cohort were previously reported in [17], which provided detailed information on infecting subtypes, timing of dual infection, and genetic distances.


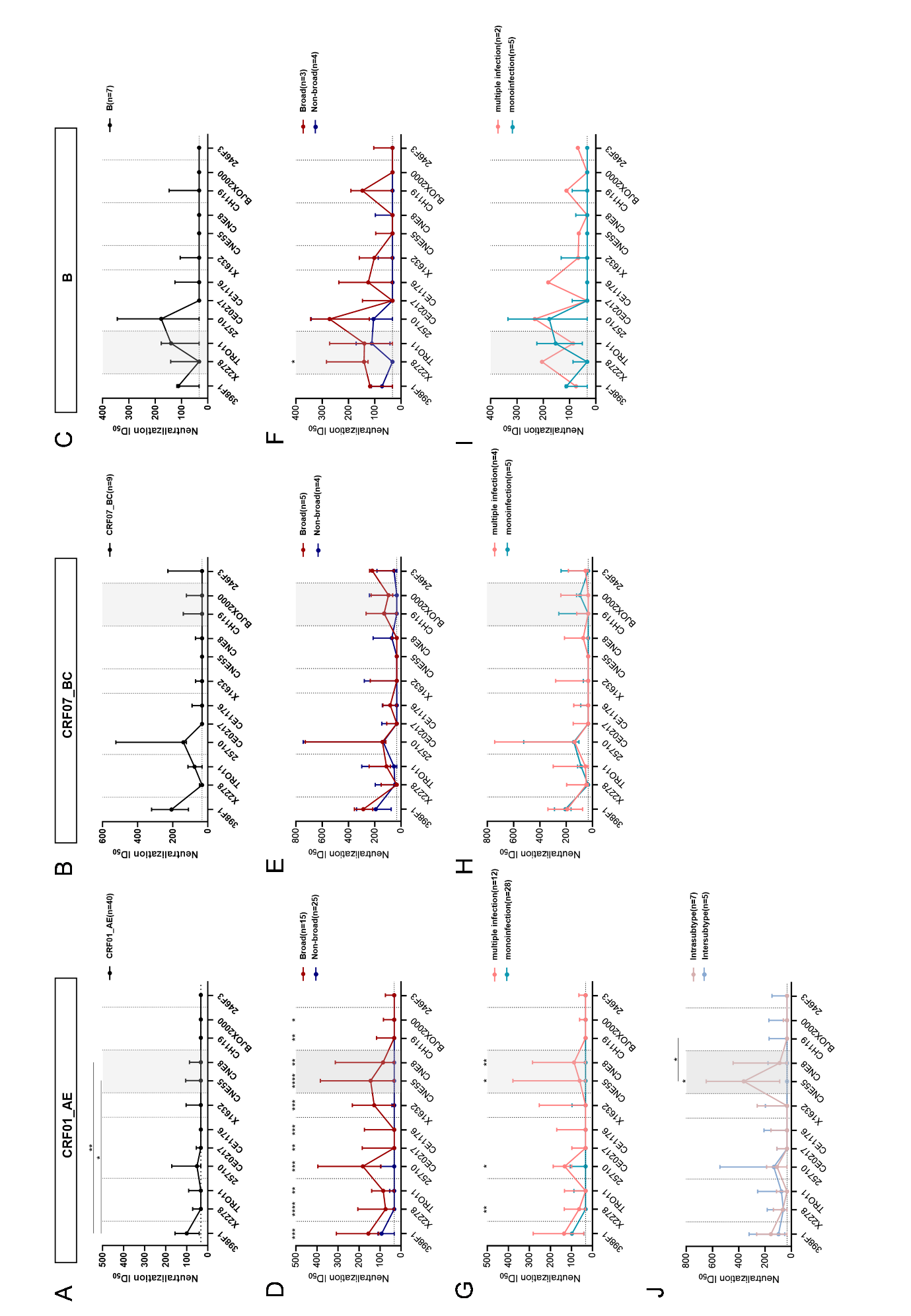


**S3 Fig. Subtype-specific neutralization against the 12-virus global panel.** Line plots show median neutralization ID₅₀ titers with interquartile ranges (IQR) from LTP plasma samples. Columns correspond to infecting subtypes: CRF01_AE, CRF07_BC, and subtype B; rows represent participant subgroups within each infecting subtype (all participants, broad versus non-broad neutralizers, and infection type [monoinfection versus multiple infection, including intrasubtype and intersubtype patterns]). Gray shading marks viruses whose subtype matches the participant’s infecting subtype. Between-group comparisons for each virus used Mann–Whitney U tests, and comparisons across viruses within the panel used Kruskal–Wallis tests. Asterisks indicate statistically significant between-subgroup differences for each virus. Horizontal bars and asterisks indicate statistically significant differences relative to the subtype-matched (shaded) viruses. Significance levels are shown above each virus (**P* < 0.05, ***P* < 0.01, ****P* < 0.001, *****P* < 0.0001). Panel I shows descriptive data for the multiple infection group (n = 2) only; no statistical testing was performed.

**
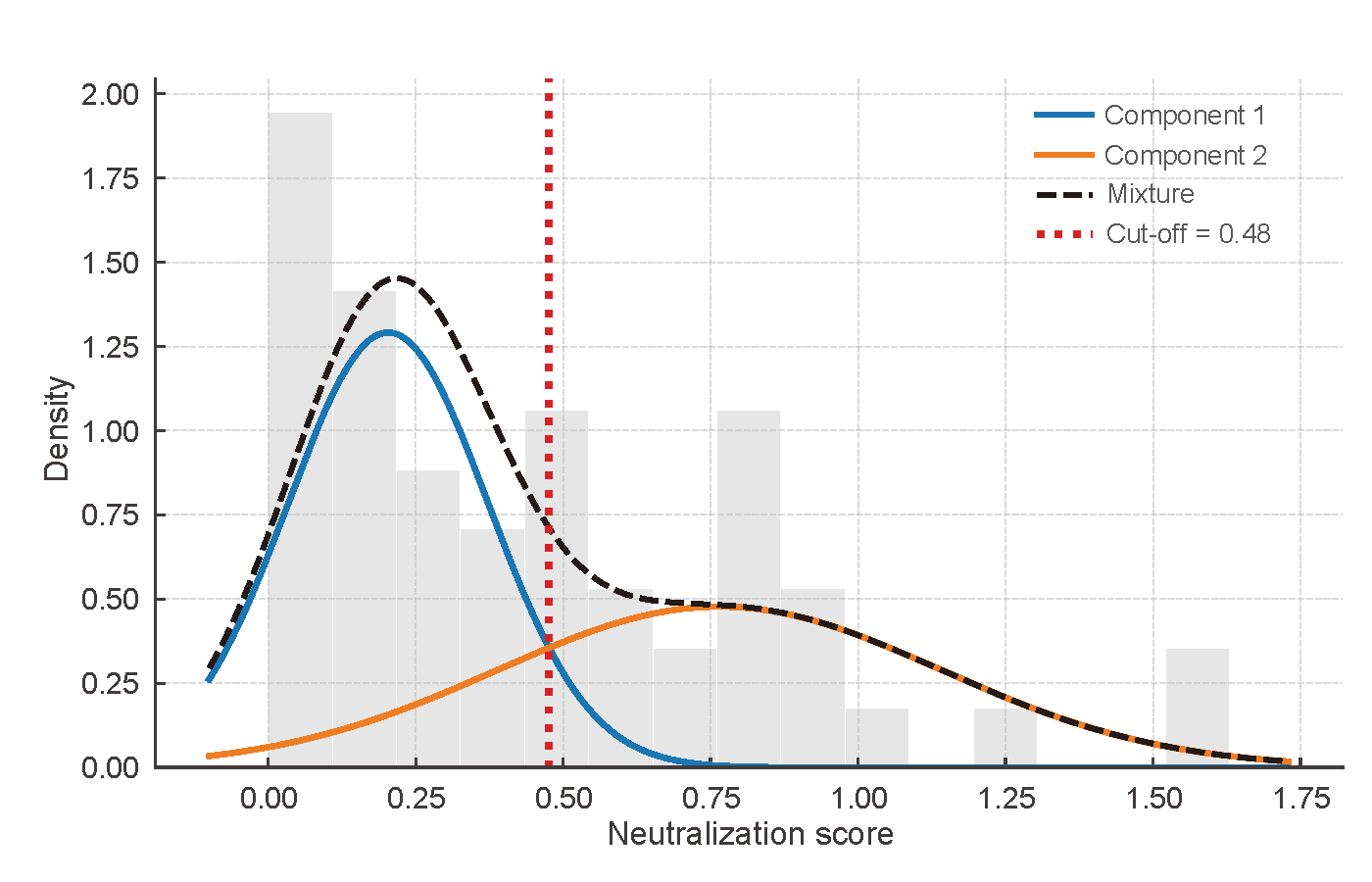
S4 Fig. Neutralization score threshold for broad neutralization derived from a two-component Gaussian mixture model (GMM).** A histogram of the neutralization scores is overlaid with a two-component GMM fit (component-specific densities and their mixture). The intersection at which the posterior probabilities are equal occurs at 0.48 (red dotted line), suggesting a bimodal distribution. Accordingly, a score > 0.48 is used as the cutoff for defining broad neutralizers, consistent with the main analyses.
