# Supplementary material for: Broadly neutralizing antibody responses in a Chinese acute HIV-1 infection cohort of men who have sex with men
Source: PLoS Pathog. 2026 Jan 2;22(1):e1013822. doi: 10.1371/journal.ppat.1013822 (PMC12758743; doi:10.1371/journal.ppat.1013822)
Supplement: S2 Text — (DOCX) [file ppat.1013822.s002.docx]

**Materials and methods**

**Viral RNA extraction and PCR**

RNA extraction, cDNA synthesis, and nested PCR were performed using the same reagents and protocols as described in previous studies [1]. Primer sequences used for the amplification of the *env* C2–V4 and *pol*-RT regions are listed in Table S1.

| **Table S1. Primers used for reverse transcription and nested PCR amplification.** | | | |
| --- | --- | --- | --- |
|  | **Primer sequence** | **Position in HXB2** | **Region** |
| **Reverse transcription primers** |  |  |  |
| 07Rev12 | 5'-AGCTGYTTRATGCCCCAGAC-3' | HXB2: 7931-7951 | *env* C2-V4 |
| rev2-1 | 5'-CTTCCTGCCATAGGAGATGCCTAA-3' | HXB2: 5957-5980 | *pol*-RT |
| **Nested PCR primers** |  |  |  |
| **Outer primers** |  |  |  |
| ED5 | 5'-ATGGGATCARAGYCTAAAGCCATGTG-3' | HXB2: 6557-6582 | *env* C2-V4 |
| 07Rev12 | 5'-AGCTGYTTRATGCCCCAGAC-3' | HXB2: 7931-7951 | *env* C2-V4 |
| MAW-26 | 5'-TGGAAATGTGGAAAGGAAGGAC-3' | HXB2: 2029-2050 | *pol*-RT |
| RT-21 | 5'-CTGTATTTCTGCTATTAAGTCTTTTGATGG-3' | HXB2: 3509-3539 | *pol*-RT |
| **Inner primers** |  |  |  |
| 07For10 | 5'-CAGTACAATGYACACATGG-3' | HXB2: 6955-6973 | *env* C2-V4 |
| ED33 | 5'-CARTAGAAAAATTCYCCTCYAC-3' | HXB2: 7357-7378 | *env* C2-V4 |
| 5-3F | 5'-GTACCAGTAAAATTAAAGCCAGGAA-3' | HXB2: 2571-2595 | *pol*-RT |
| 5-3R | 5'-GGCTCTAAGATTTTTGTCATGCT-3' | HXB2: 3036-3058 | *pol*-RT |

**Next-generation sequencing and bioinformatics analysis**

Amplicons were purified with Agencourt AMPure XP beads (Beckman Coulter, USA), quantified using the Qubit dsDNA BR Assay Kit (Life Technologies, USA), and quality-checked using the Agilent 2100 Bioanalyzer (Agilent Technologies, USA). Libraries were prepared following the TruSeq Nano DNA Library Prep Reference Guide and sequenced on the Illumina MiSeq platform (Illumina, USA). Raw reads were trimmed, filtered (Q-score < 30, length < 350 bp, or frequency < 1%), and aligned to reference sequences using Geneious (v2021.2.2). HIV-1 multiple infection was identified based on neighbor-joining phylogenetic trees, genetic distances, and highlighter analysis [2].

**Neutralization assays**

HIV-1 pseudoviruses were produced by co-transfecting HEK293T cells with the pNL4-3.Luc.E(-)R(-) backbone vector and each envelope expression plasmid using jetPRIME reagent (Polyplus, France). Pseudovirus-containing supernatants were harvested 48 hours post-transfection. Pseudovirus titration and neutralization assays were performed in TZM-bl cells as previously described [3–5]. To assess viral infectivity, pseudoviruses were subjected to 5-fold serial dilutions (7 steps) in quadruplicate in 96-well plates (100 μl/well). TZM-bl cells (15,000 cells/well) in 100 μl of growth medium containing 30 μg/ml DEAE-dextran were then added and incubated at 37 °C in 5% CO₂ for 48 hours. Cell-only wells were included as negative controls. Luminescence was measured using the Bright-Glo Luciferase Assay System (Promega, USA) on a SPARK multimode plate reader (Tecan, Switzerland). Wells with luminescence values exceeding three times the background were considered positive, and 50% tissue culture infectious dose (TCID₅₀) values were calculated accordingly. For neutralization assays, heat-inactivated plasma (56 °C for 1 hour) samples were serially diluted 3-fold (7 steps, starting at 1:50) in triplicate in 96-well plates. Pseudoviruses (200 TCID₅₀) were then added, and the plasma–virus mixtures (150 μl/well) were incubated at 37 °C for 1 hour. Virus-only wells were included as infection controls. TZM-bl cells (15,000 cells/well) in 100 μl of growth medium containing 37.5 μg/ml DEAE-dextran were then added and incubated at 37 °C in 5% CO₂ for 48 hours. Neutralization activity was calculated as the percentage reduction in luminescence relative to virus-only wells. The 50% inhibitory dilution (ID₅₀) values were determined by nonlinear regression.

**Calculation of neutralization score**

Neutralization breadth and potency were quantified using a composite neutralization score derived from the ID_50_ values across a standardized 12-virus panel, calculated as the average of log-transformed ID_50_ values [score = average (log3 (dilution/100) + 1)]. Titers below the detection limit (1:50) were assigned a value of 33 for score calculation, with a log-transformed value of 0.0 indicating undetectable neutralization [6–8].

**Statistical analyses**

Broad neutralization was defined as a neutralization score ≥ 0.5, and this binary outcome was modeled using logistic regression to identify factors associated with bnAb responses (R v4.4.0). Group comparisons were performed using Fisher’s exact test, Student’s t-test, the Mann–Whitney U test, or the Kruskal–Wallis test, as appropriate (IBM SPSS Statistics v26.0). Missing data were multiply imputed using the mice package, and data visualization was conducted using GraphPad Prism (v10.1.2) and the ggplot2 package. Statistical significance was defined as *P* < 0.05. To assess the robustness of our findings, we conducted a sensitivity analysis by fitting a two-component Gaussian mixture model (GMM) to the neutralization scores to derive a data-driven threshold distinguishing broad from non-broad neutralization.

**Supplementary references**

1. Sun X, Zhang H, Kong X, Li N, Zhang T, An M, et al. Low-level viremia episodes appear to affect the provirus composition of the circulating cellular HIV reservoir during antiretroviral therapy. Front Microbiol. 2024 May 22;15:1376144.

2. Luan H, Han X, Yu X, An M, Zhang H, Zhao B, et al. Dual Infection Contributes to Rapid Disease Progression in Men Who Have Sex With Men in China. JAIDS Journal of Acquired Immune Deficiency Syndromes. 2017 Aug 1;75(4):480–7.

3. deCamp A, Hraber P, Bailer RT, Seaman MS, Ochsenbauer C, Kappes J, et al. Global Panel of HIV-1 Env Reference Strains for Standardized Assessments of Vaccine-Elicited Neutralizing Antibodies. Hahn BH, editor. J Virol. 2014 Mar;88(5):2489–507.

4. Li M, Gao F, Mascola JR, Stamatatos L, Polonis VR, Koutsoukos M, et al. Human Immunodeﬁciency Virus Type 1 env Clones from Acute and Early Subtype B Infections for Standardized Assessments of Vaccine-Elicited Neutralizing Antibodies. J VIROL. 2005;79.

5. Molinos-Albert LM, Baquero E, Bouvin-Pley M, Lorin V, Charre C, Planchais C, et al. Anti-V1/V3-glycan broadly HIV-1 neutralizing antibodies in a post-treatment controller. Cell Host & Microbe. 2023 Aug;31(8):1275-1287.e8.

6. Landais E, Huang X, Havenar-Daughton C, Murrell B, Price MA, Wickramasinghe L, et al. Broadly Neutralizing Antibody Responses in a Large Longitudinal Sub-Saharan HIV Primary Infection Cohort. Trkola A, editor. PLoS Pathog. 2016 Jan 14;12(1):e1005369.

7. Granger LA, Huettner I, Debeljak F, Kaleebu P, Schechter M, Tambussi G, et al. Broadly neutralizing antibody responses in the longitudinal primary HIV-1 infection Short Pulse Anti-Retroviral Therapy at Seroconversion cohort. AIDS. 2021 Nov 1;35(13):2073–84.

8. Simek MD, Rida W, Priddy FH, Pung P, Carrow E, Laufer DS, et al. Human Immunodeficiency Virus Type 1 Elite Neutralizers: Individuals with Broad and Potent Neutralizing Activity Identified by Using a High-Throughput Neutralization Assay together with an Analytical Selection Algorithm. J Virol. 2009 July 15;83(14):7337–48.
